# Task-Specific Rescue Loading Redistributes Lower Limb Joint Work During Simulated Sprinting Across Fatigue States in Firefighters

**DOI:** 10.3390/life16071123

**Published:** 2026-07-06

**Authors:** Junqiang Zhang, Xueyi Chen, Min Chen, Xinxin Zhang

**Affiliations:** 1 School of Physical Education, Shaanxi Normal University, Xi’an 710119, China; shiyan2024@snnu.edu.cn (J.Z.); xueyi2003@snnu.edu.cn (X.C.); 2 School of Physical Education and Art, East China University of Technology, Nanchang 330013, China; 3 College of Physical Education and Health, Guangxi Normal University, Guilin 541006, China

**Keywords:** biomechanics, fatigue, firefighter, joint work, load carriage, rescue sprinting

## Abstract

Firefighters routinely perform short-distance sprints while wearing or carrying occupational gear. Nevertheless, how fatigue interacts with task-specific loading to shape the distribution of mechanical work across lower-limb joints remains poorly understood. This study aimed to determine how fatigue state and task-specific rescue loading affect sprint performance, impact loading, lower-limb joint mechanics, joint work contribution, and muscle activation in male firefighters. Fifty-three male firefighters completed four simulated rescue sprinting tasks under five fatigue conditions: non-fatigue, whole-body fatigue, and mild, moderate, and severe local knee extensor fatigue. Tasks included unloaded sprinting and 7, 20, and 30 kg task-specific rescue-load conditions. Kinematic, kinetic, and electromyographic data were collected during the stance phase of the dominant limb. Mild local fatigue was associated with a sprinting speed comparable to the non-fatigue condition and higher than that under whole-body or moderate-to-severe local fatigue. Progressively heavier task-specific rescue loads decreased sprinting speed, prolonged stance time, increased knee work contribution, and decreased ankle work contribution, indicating a redistribution of stance-phase mechanical work from the ankle toward the knee. This compensatory pattern was further modulated by treadmill-induced global locomotor fatigue. In conclusion, this ankle-to-knee redistribution may help firefighters complete high-load rescue sprinting, but it may also increase knee mechanical demand, particularly under global locomotor fatigue.

## 1. Introduction

Firefighters constitute a distinct category of occupational athletes. They must execute high-intensity rescue tasks under considerable time pressure while carrying task-specific loads and responding to unpredictable movement demands. Musculoskeletal disorders are prevalent in this population, and both physical fitness and musculoskeletal health are closely linked to occupational performance and injury risk [1,2]. Clinical perspectives further identify fatigue management, movement competence, recovery strategies, and overexertion as modifiable risk factors [3]. In addition, personal protective equipment and task-specific gear can impair mobility, alter gait mechanics, and compromise dynamic balance, thereby compounding the mechanical demands of rescue locomotion [4]. Consequently, a deeper understanding of how firefighters regulate lower limb mechanics during loaded rapid locomotion has direct implications for performance optimization and injury prevention.

Rescue sprinting in firefighters differs from conventional load-carriage running because the carried objects vary in mass, shape, placement, and handling technique. Previous firefighter-specific studies have shown that simulated rescue tasks reduce locomotor speed, prolong stance time, and alter lower-limb kinematics, kinetics, dynamic stability, and work performance [5,6]. These findings indicate that rescue-load sprinting should be considered a task-specific locomotor condition rather than a simple extension of conventional running or symmetric load carriage. Although recent biomechanical studies have demonstrated that manipulating locomotor loads and using sport-specific sprint protocols can reveal adaptations that may be missed by generic laboratory assessments [7,8,9], most load-carriage research has focused on military-style symmetric loads or isolated load conditions [10,11,12,13,14,15,16]. In contrast, firefighting rescue tasks often involve asymmetric hand-carried or shoulder-borne loads performed under time pressure. Such tasks require not only lower-limb propulsion and impact attenuation, but also gripping, object stabilization, and trunk control. Accordingly, examining lower-limb stance mechanics and joint work redistribution under task-specific rescue loads may help clarify how firefighters regulate mechanical demand while maintaining whole-body load control during rescue sprinting.

Fatigue represents another critical factor that can nonlinearly modulate rescue sprint performance. Exercise-induced fatigue reduces neuromuscular force output, impairs proprioception, and degrades movement control, thereby affecting stance mechanics, impact attenuation, and push-off propulsion [17]. Mild local fatigue or preactivation may transiently enhance subsequent explosive performance when potentiation-like effects outweigh fatigue-related inhibition. In contrast, whole-body fatigue and more severe local fatigue are expected to impair locomotor capacity [18,19]. Subdividing fatigue into nonfatigue, whole-body fatigue, and graded local knee extensor fatigue thus helps clarify the transition from short-term activation to functional inhibition. From a multijoint mechanical perspective, both task-specific rescue loads and fatigue may reorganize the distribution of lower-limb work. As task-specific loading increases or distal function deteriorates, mechanical work may shift from the ankle toward the knee or hip to sustain task completion [10,11,20]. Joint work contribution analysis provides a useful framework for identifying compensatory strategies, as it quantifies how mechanical demand is redistributed across joints during stance [21,22]. Whether this redistribution occurs during firefighter rescue sprinting, and whether fatigue state, particularly whole-body fatigue, modulates the compensatory demand on the knee, remains unclear.

Recent firefighter-specific studies, including those by Mao et al. [23] and Iwańska et al. [24], have advanced understanding of fatigue-, load-, and environment-related changes in sprinting and lower-limb biomechanics. However, these studies did not specifically quantify how stance-phase mechanical work is redistributed among the hip, knee, and ankle during object-specific rescue sprinting, nor did they examine whether fatigue state modulates this joint work-contribution pattern. Building on this gap, the present study aimed to determine how fatigue state and task-specific rescue loading affect sprint performance, impact loading, lower-limb joint mechanics, joint work contribution, and muscle activation in male firefighters. This investigation hypothesized that mild local fatigue would transiently improve sprint performance, whereas whole-body fatigue and moderate-to-severe local fatigue would impair it. An additional hypothesis stated that progressively heavier task-specific rescue loads would shift joint work contribution from the ankle toward the knee, and that fatigue state, especially treadmill-induced global locomotor fatigue, would modulate the magnitude of this task-related redistribution.

## 2. Materials and Methods

### 2.1. Participants

An a priori sample size estimation was performed using G*Power 3.1, assuming an effect size of 0.25, an alpha level of 0.05, and a statistical power of 0.95. This calculation indicated that at least 32 participants were required [25]. Fifty-three male firefighters were recruited from the Guilin Fire and Rescue Detachment (Table 1). Eligibility required that participants be active firefighters with regular firefighting training, free from lower limb musculoskeletal injury in the preceding year, and capable of completing the fatigue induction and rescue sprinting protocols. Exclusion criteria included cardiovascular, neurological, or vestibular disorders; use of medication that could influence neuromuscular function or performance; and any pain or discomfort during testing. All participants provided written informed consent. This study received approval from the Ethics Committee of Guangxi Normal University (20230419001).

### 2.2. Experimental Design and Rescue Tasks

**Figure 1:**
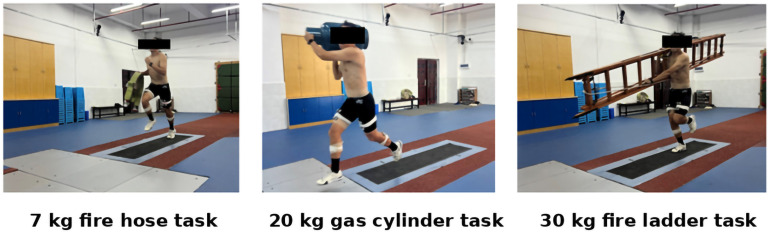
Schematic illustration of the three loaded simulated rescue sprinting tasks.

A 5 × 4 repeated-measures design was employed. Participants wore standardized athletic clothing and running shoes rather than full firefighting protective clothing, because reflective markers and surface EMG electrodes required direct access to anatomical landmarks and skin. The five fatigue states included non-fatigue, whole-body fatigue, and mild, moderate, and severe local knee-extensor fatigue. The four rescue tasks comprised unloaded sprinting, sprinting while carrying a 7 kg fire hose, a 20 kg gas cylinder, or a 30 kg fire ladder. In accordance with firefighting practice, the gas cylinder and fire ladder were carried on one shoulder, whereas the hose was hand-carried. Figure 1 illustrates the three simulated rescue sprinting tasks. Testing sessions for different fatigue states were separated by at least 48 h, and the order of both fatigue states and tasks was randomized. For each fatigue state, all dynamic rescue sprinting trials were conducted as soon as possible after the fatigue criterion was met. Three successful trials were collected per task, and the average value was used for analysis.

### 2.3. Experimental Procedure and Fatigue Protocols

**Figure 2:**
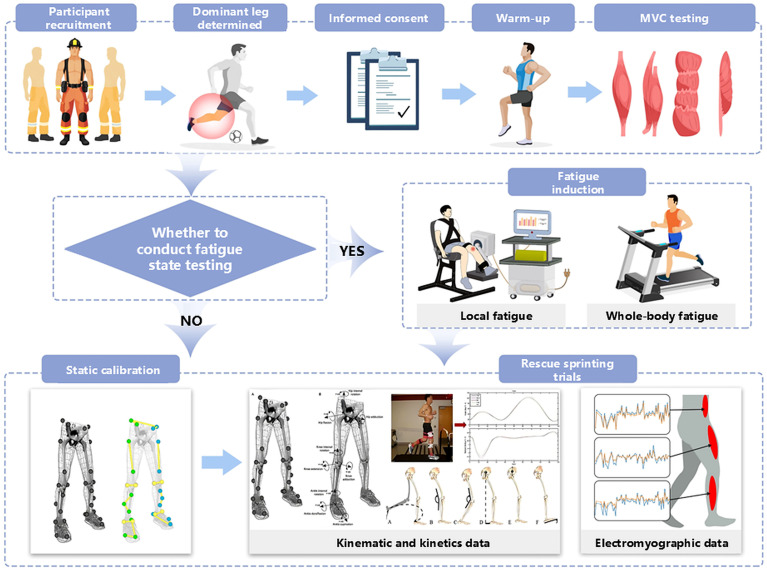
Experimental flowchart showing warm-up, MVC testing, fatigue induction, static calibration, and dynamic rescue sprinting trials.

The testing procedure consisted of preparation, maximum voluntary contraction (MVC) testing, fatigue induction, static calibration, and dynamic rescue sprinting. Participants first completed a standardized warm-up comprising 5 min of jogging and 5 min of lower-limb dynamic stretching. Whole-body fatigue was operationally defined as treadmill-induced global locomotor fatigue during running-based rescue sprinting. Participants first ran at 5 km/h and 0% grade for 14 min. Thereafter, the treadmill grade increased by 2% every 2 min to 14%, followed by incremental speed increases of 0.5 km/h each minute. The protocol was terminated when participants reached at least 90% of their predicted maximal heart rate and could no longer maintain the required running speed despite verbal encouragement. Local knee extensor fatigue was induced on the dominant limb using an isokinetic dynamometer. After familiarization and a baseline assessment of maximal knee-extension torque at 60 deg/s, participants repeatedly performed maximal knee extensions. Mild, moderate, and severe local fatigue were defined as three consecutive peak knee-extension torques decreasing by 25%, 50%, and 75% from baseline, respectively. The experimental workflow is shown in Figure 2.

### 2.4. Data Collection

A trained research team conducted data collection. One investigator operated the motion-capture and force-platform systems, one investigator managed EMG preparation and signal monitoring, one investigator supervised the fatigue protocol and safety monitoring, and one investigator provided standardized instructions and checked trial validity. All investigators were trained before formal testing to ensure consistency in marker placement, EMG electrode placement, fatigue induction, and trial acceptance criteria. Three-dimensional marker trajectories were collected using an eight-camera Qualisys motion-capture system (Oqus600+, Qualisys AB, Göteborg, Sweden) at 200 Hz. Ground reaction forces were recorded simultaneously using one Kistler force platform (9287CAQ10, Kistler, Winterthur, Switzerland) and one AMTI force platform (BP4602070RS-2K, Advanced Mechanical Technology Inc., Watertown, MA, USA) at 2000 Hz. Surface electromyography (EMG) was collected from the dominant-side gluteus maximus, rectus abdominis, rectus femoris, biceps femoris, gastrocnemius, and tibialis anterior using a Noraxon wireless system (TeleMyo 2400T, Noraxon, Scottsdale, AZ, USA) at 2000 Hz. Marker trajectories, force-plate data, and EMG signals were synchronized through Qualisys Track Manager. EMG electrodes were placed according to SENIAM recommendations [26]. For each muscle, three 5 s MVC trials were recorded with 60 s rest between trials, and the maximum 1 s root mean square (RMS) value was used for EMG normalization [27]. Before electrode placement, the skin was shaved when necessary, lightly abraded, and cleaned with alcohol to reduce skin impedance. Electrodes were attached after the skin was dry, and signal quality was visually checked before MVC and dynamic trials. Upper-limb and posterior trunk-extensor EMGs were not recorded because the primary biomechanical model and hypotheses focused on lower-limb stance mechanics and joint work contributions.

### 2.5. Data Processing and Outcome Variables

The analysis focused on the stance phase of the dominant limb, as this period captures impact attenuation, body support, and push off. Stance was defined as the interval from dominant foot contact to toe off, using a vertical ground reaction force threshold of 10 N [28]. A three-dimensional model was constructed in Visual3D (C-Motion Inc., Germantown, MD, USA). Kinematic data were filtered using a fourth-order low-pass Butterworth filter with a 14 Hz cutoff frequency. Kinetic data were filtered using a fourth-order low-pass Butterworth filter with a 100 Hz cutoff frequency. EMG signals were band-pass filtered from 10 to 500 Hz, full-wave rectified, and processed to obtain stance-phase RMS values normalized to MVC.

The primary outcomes included stance time, sprinting speed, peak vertical ground reaction force (vGRF), peak knee extension moment, hip/knee/ankle joint work contribution, and RMS amplitudes of the rectus femoris and rectus abdominis. These variables directly reflected sprint performance, impact loading, knee mechanical demand, joint work redistribution, and neuromuscular compensation. Secondary outcomes comprised step width, sagittal-plane peak joint angles of the hip, knee, and ankle, center of mass (CoM) displacement in the mediolateral and vertical directions, other sagittal-plane peak joint moments, and RMS amplitudes of the remaining recorded muscles. All kinetic outcomes were normalized to body mass. Joint work calculations followed inverse dynamics principles, as expressed in Equations (1)–(5).

(1) $$M_{noram}=\frac{M_{joint}}{m}$$

(2) $${vGRF}_{norm}=\frac{F_{zmax}}{m}$$

(3) $$P_{j}\left(t\right)=M_{j}\left(t\right)\times \omega_{j}\left(t\right)$$

(4) $$W_{j}=\int_{t1}^{t2}\left|P_{j}\left(t\right)\right|dt$$

(5) $${Cont}_{j}=\frac{\left|W_{j}\right|}{\left|W_{hip}\right|+\left|W_{knee}\right|+\left|W_{ankle}\right|}$$

where j denotes the hip, knee, or ankle joint; $M_{joint}$ is the net joint moment; m is body mass; $M_{noram}$ is the body-mass-normalized joint moment; $F_{zmax}$ is the peak vertical ground reaction force; ${vGRF}_{norm}$ is the body-mass-normalized peak vGRF; $\omega_{j}\left(t\right)$ is joint angular velocity; $P_{j}\left(t\right)$ is joint power; t1 and t2 represent initial contact and toe-off, respectively; $W_{j}$ is the absolute mechanical work of joint j during stance; and ${Cont}_{j}$ is the joint work contribution. Joint work contribution is a unitless proportion ranging from 0 to 1, and the sum of the hip, knee, and ankle contributions equals 1.

### 2.6. Statistical Analysis

All statistical analyses were conducted using SPSS 25.0. Normality was checked with the Shapiro–Wilk test, and sphericity was evaluated using Mauchly’s test. When sphericity was violated, the Greenhouse–Geisser correction was applied. A two-way 5 × 4 repeated-measures ANOVA was employed to examine the main effects of fatigue state and rescue task, as well as their interaction, for each outcome. Bonferroni adjusted post hoc tests were used where appropriate. Statistical significance was set at *p* < 0.05. Effect sizes are reported as partial eta squared (ηp^2^), derived from the F statistics and corresponding degrees of freedom. Greenhouse–Geisser-corrected *p*-values are reported whenever the sphericity assumption is violated [29].

## 3. Results

Descriptive statistics for the primary outcomes are presented in Table 2 and Table 3, whereas the ANOVA results for all outcomes discussed in this section are summarized in Table 4.

### 3.1. Sprint Performance and Key Spatiotemporal Outcomes

Both fatigue state and rescue task, as well as their interaction, significantly influenced stance time and sprinting speed (Table 4). Mild local fatigue produced a slightly shorter stance time and a sprinting speed comparable to the non-fatigue condition but higher than that observed under whole-body fatigue or moderate to severe local fatigue (Table 3). Across progressively heavier task-specific rescue conditions, sprinting speed steadily declined from 6.112 ± 0.419 m/s during unloaded sprinting to 4.871 ± 0.321 m/s during the 30 kg ladder task. Stance time concurrently increased from 0.138 ± 0.013 s to 0.199 ± 0.018 s (Table 2). Step width showed a smaller task effect but a significant fatigue-by-task interaction, suggesting that balance adjustments during loaded sprinting depend on the specific combination of fatigue and load (Table 4).

### 3.2. Joint Kinematics, Center-of-Mass Displacement, and Impact Loading

**Figure 3:**
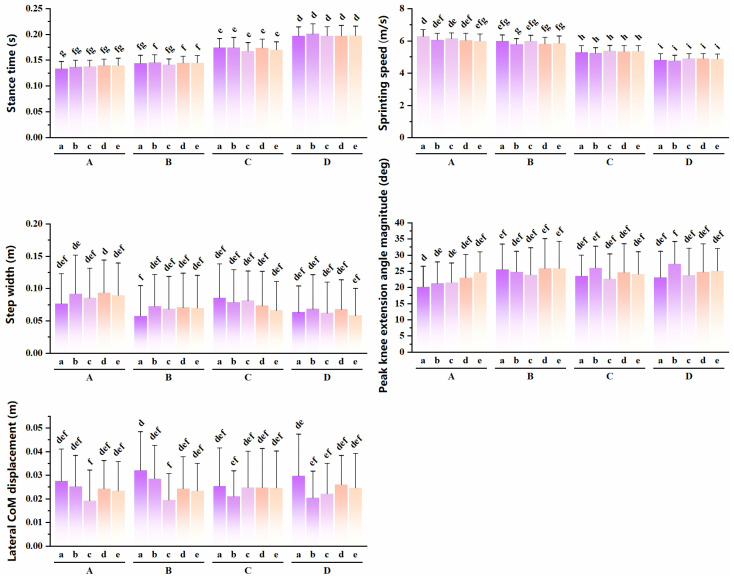
Interaction effects for key spatiotemporal and kinematic outcomes: (A) unloaded sprinting; (B) 7 kg fire hose; (C) 20 kg gas cylinder; (D) 30 kg fire ladder; (a) non-fatigue; (b) whole-body fatigue; (c) mild local fatigue; (d) moderate local fatigue; (e) severe local fatigue; and (CoM) center of mass. The different lowercase letters above the bars indicate Bonferroni-adjusted pairwise differences among fatigue states within each rescue-task condition.

Supporting kinematic analyses indicated that the rescue-task condition altered lower-limb stance mechanics, with significant task effects observed for peak hip flexion, knee flexion, and ankle dorsiflexion (Table 4). These findings suggest a more flexed sagittal-plane stance posture during loaded rescue sprinting, likely reflecting a strategy to increase the range and time available for impact attenuation while maintaining forward progression. Center-of-mass displacement showed direction-specific responses. Vertical CoM displacement was significantly affected by both fatigue state and rescue task, but no fatigue × task interaction was detected. By contrast, lateral CoM displacement showed a significant fatigue × task interaction, suggesting that mediolateral body-control demands were more sensitive to combined fatigue and task conditions. Peak knee extension angle exhibited a fatigue × task interaction, implying that sagittal-plane knee posture was sensitive to combined fatigue-load conditions (Table 4). Regarding impact loading, peak vGRF was significantly affected by the rescue task and by the fatigue × task interaction (Table 4). Specifically, the peak vGRF increased from 28.106 ± 2.955 N/kg during unloaded sprinting to 30.911 ± 5.390 N/kg during the 30 kg fire-ladder task (Table 2). Thus, the reduction in sprinting speed and the prolongation of stance time during heavier tasks did not fully offset the increase in vertical impact loading. The interaction patterns for key spatiotemporal and kinematic outcomes are illustrated in Figure 3.

### 3.3. Joint Moments and Joint Work Contribution

**Figure 4:**
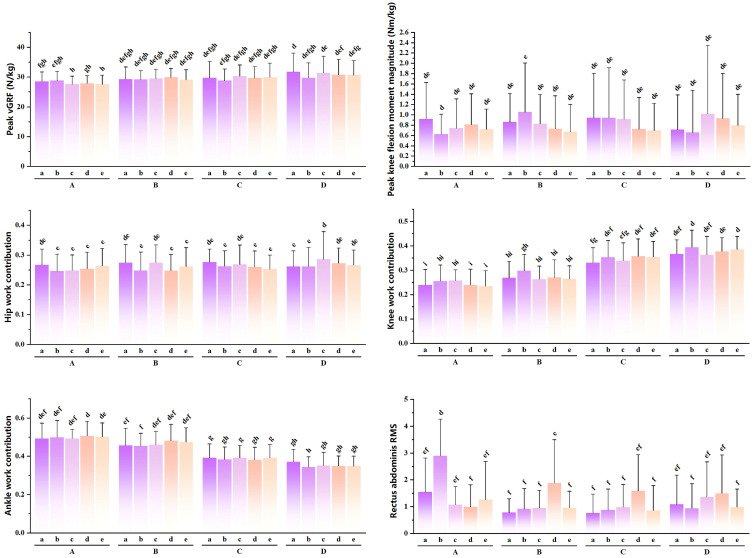
Interaction effects for kinetic outcomes, joint work contribution, and rectus abdominis RMS amplitude: (A) unloaded sprinting; (B) 7 kg fire hose; (C) 20 kg gas cylinder; (D) 30 kg fire ladder; (a) non-fatigue; (b) whole-body fatigue; (c) mild local fatigue; (d) moderate local fatigue; (e) severe local fatigue; (vGRF) vertical ground reaction force; and (RMS) root mean square. The different lowercase letters above the bars indicate Bonferroni-adjusted pairwise differences among fatigue states within each rescue-task condition.

Joint moment outcomes supported a task-specific elevation in knee mechanical demand. The peak knee extension moment increased from 3.98 ± 0.70 Nm/kg during unloaded sprinting to 4.71 ± 0.70 Nm/kg during the 30 kg ladder task (Table 2; ANOVA results in Table 4). Although the peak knee extension moment did not show a fatigue-by-task interaction, the magnitude of the peak knee flexion moment did exhibit a significant interaction effect, suggesting that knee joint moment regulation varies across different fatigue-task combinations. More importantly, a task-related redistribution of stance-phase joint work contributions emerged. Knee work contribution rose from 0.245 ± 0.061 in the unloaded condition to 0.377 ± 0.064 in the 30 kg ladder task, whereas ankle work contribution fell from 0.499 ± 0.074 to 0.353 ± 0.059 (Table 2). The contribution of hip work changed only modestly across tasks. Significant fatigue-by-task interactions were also detected for hip, knee, and ankle work contributions (Table 4). Of note, whole-body fatigue was associated with a higher knee work contribution than the non-fatigue condition (Table 3). Taken together, these results indicate that progressively heavier task-specific rescue loads shift mechanical work contribution from the ankle toward the knee, and that whole-body fatigue may further increase the compensatory demand on the knee. Figure 4 illustrates the interaction patterns for peak vGRF, peak knee flexion moment magnitude, and joint work contribution.

### 3.4. Muscle Activation

Muscle activation data provided neuromuscular support for the observed redistribution of joint work. The rectus femoris RMS increased from 1.489 ± 1.078 during unloaded sprinting to 2.308 ± 1.755 during the 30 kg fire-ladder task (Table 2), consistent with greater knee extension moment and higher knee work contribution under heavier task-specific loading. However, the rectus femoris RMS showed no fatigue × task interaction. In contrast, the rectus abdominis RMS was lower during loaded tasks than during unloaded sprinting and exhibited a significant fatigue × task interaction (Table 2 and Table 4), suggesting that asymmetric load carriage may have altered trunk-control demands. The interaction pattern for rectus abdominis RMSs is shown in Figure 4. Activation of the remaining recorded muscles was not emphasized because these data were not central to interpreting the joint work redistribution pattern.

## 4. Discussion

The present study examined the combined effects of fatigue state and a rescue task on biomechanics during simulated rescue sprinting in firefighters. Several key findings emerged. First, mild local knee-extensor fatigue was associated with sprinting speed comparable to that under the non-fatigue condition and higher than that observed under whole-body and moderate-to-severe local fatigue, along with a slightly shorter stance time. Second, progressively heavier task-specific rescue conditions induced a load-attenuation strategy characterized by reduced speed, prolonged stance time, and greater lower-limb flexion. Yet, this strategy did not fully prevent an increase in vertical ground reaction force during the 30 kg fire-ladder task. Most importantly, task-specific rescue loading shifted mechanical work contribution from the ankle toward the knee, and whole-body fatigue was associated with greater knee work contribution overall. These findings support the concept that firefighters employ a distal-to-knee shift in joint work contribution when performing high-load rescue sprinting.

### 4.1. Staged Effects of Fatigue on Rescue Sprint Performance

The finding that mild local fatigue produced a slightly higher sprint speed and shorter stance time than more severe fatigue conditions indicates that fatigue does not impair rescue sprint performance in a strictly monotonic manner. Mild local fatigue, induced by repeated knee extensor contractions, may have served as a short-term activation stimulus. This interpretation aligns with the view that subsequent explosive performance depends on the balance between potentiation-like enhancement and fatigue-related inhibition [18,19]. When the activation effect predominates, short-duration sprint performance may be maintained or slightly enhanced. As fatigue becomes more severe, however, force-generating capacity, motor unit recruitment, proprioception, and interjoint coordination tend to deteriorate, leading to a reduced sprinting speed and prolonged stance time [17]. Whole-body fatigue may further impair performance by imposing combined cardiovascular, metabolic, and neuromuscular constraints. Distinguishing fatigue states is therefore essential when interpreting rescue sprint performance. Because the rectus femoris RMS did not increase under mild local fatigue, this apparent performance advantage should not be interpreted as a simple increase in quadriceps activation. It may instead reflect transient neuromuscular readiness, interjoint coordination, or contributions from unmeasured synergistic muscles.

### 4.2. Load-Induced Flexed Stance Strategy and Impact Loading

Across progressively heavier task-specific rescue conditions from unloaded sprinting to hose, gas-cylinder, and ladder carriage, the firefighters progressively reduced their sprinting speed and increased their stance time. Simultaneously, peak hip flexion, knee flexion, and ankle dorsiflexion increased, indicating a more flexed sagittal-plane stance posture. This pattern can be interpreted as a load-attenuation strategy, in which the firefighters slow down, increase the time available for impact attenuation, and enlarge the joint motion range to absorb loads. Similar load-management principles have been observed in firefighter rescue tasks, lower-body positive pressure treadmill running, and load-carriage running [5,7,10,11,12,13,14,15,16]. However, the 30 kg fire-ladder task still produced the highest vertical ground reaction force, demonstrating that the combined strategy of reducing speed and increasing stance time only partially offsets the mechanical consequences of heavier task-specific rescue loading. Thus, the mechanical demands of high-load rescue sprinting appear to arise not from excessive speed, but rather from the combined increases in task-specific rescue loading, stance duration, and impact-attenuation requirements. Therefore, slower sprinting under heavier task-specific rescue loading should not be interpreted as lower mechanical demand.

### 4.3. Distal-to-Knee Redistribution of Joint Work and Neuromuscular Mechanism

The most important biomechanical finding was the redistribution of joint work contribution from the ankle toward the knee across progressively heavier task-specific rescue conditions. During unloaded sprinting, the ankle contributed approximately half of the total lower-limb stance-phase work, consistent with its role in propulsion and elastic energy return. During the 30 kg fire-ladder task, ankle contribution decreased while knee contribution increased substantially. The magnitude of the task effect was particularly large for contributions from knee and ankle work, indicating that task-specific rescue loading was the dominant factor associated with this redistribution. Similar distal-to-proximal shifts have been reported during load carriage and prolonged running, and joint-energetics research underscores the importance of examining how work is distributed across joints rather than relying solely on global ground-reaction-force measures [10,11,20,21]. The present study extends this concept to simulated firefighting rescue tasks involving asymmetric and object-specific loads. The task effect remained the dominant factor associated with the ankle-to-knee redistribution, whereas fatigue appeared to modulate the magnitude of this compensatory pattern. Fatigue may modulate ankle-to-knee redistribution because treadmill-induced global locomotor fatigue can reduce force-generating capacity, proprioceptive control, and interjoint coordination. Under these conditions, firefighters may rely more heavily on the knee to regulate stance stability, impact attenuation, and forward propulsion when ankle contribution decreases.

Notably, the redistribution was expressed mainly as an increase in knee contribution rather than a marked increase in hip contribution. This pattern may be related to the rapid stance transitions required during rescue sprinting. Under asymmetric hand- or shoulder-borne loading, trunk and hip motion may be constrained by the need to stabilize the carried object, whereas the knee can act as an intermediate joint for impact attenuation, forward propulsion, and rapid adjustment of the center of mass. The concurrent increase in knee extension moment and rectus femoris activation supports this interpretation.

This compensatory redistribution carries both functional and potential risk implications. Functionally, increasing knee contribution may help firefighters maintain stance stability, impact attenuation, and forward propulsion when ankle contribution diminishes under heavy task-specific rescue loading conditions. However, when high-load tasks are performed under whole-body fatigue, the knee may face both a greater work contribution and increased vertical impact loading. Such combined mechanical demands could increase the regulatory burden on the knee joint, particularly when high-load rescue sprints are repeated frequently or performed with inadequate recovery. Therefore, the distal-to-knee shift should be viewed as task-preserving compensation rather than a purely beneficial adaptation. The electromyography results provide additional insight into this compensation. Rectus femoris activation increased during the 20 kg gas-cylinder and 30 kg fire-ladder tasks, consistent with a greater knee-extensor demand. In contrast, rectus abdominis activation was lower during the loaded tasks than during unloaded sprinting. Because the rescue loads were asymmetric and typically shoulder- or hand-carried, trunk and upper-limb control should also be considered. Shoulder-borne and hand-carried loads likely require coordinated activation of the erector spinae, latissimus dorsi, shoulder stabilizers, and gripping muscles to stabilize the trunk and object. Therefore, the observed lower-limb work redistribution may have occurred together with unmeasured trunk and upper-limb compensations. This explanation remains speculative because only the rectus abdominis was recorded among the trunk muscles, and upper-limb muscles were not measured. Future work should include multi-channel trunk and upper-limb electromyography to clarify whole-body load-control strategies under asymmetric rescue-load conditions.

### 4.4. Practical Implications

The current findings have practical implications for firefighter conditioning and rescue-task training. Evaluating rescue sprint performance solely by completion speed may underestimate mechanical demand, because the 30 kg ladder task produced higher impact loading and greater knee mechanical demand despite a reduction in sprinting speed. The observed shift in joint work contribution from the ankle to the knee suggests that high-load rescue sprinting requires not only task-completion capacity but also adequate knee-load tolerance and lower-limb coordination. Therefore, training programs should emphasize progressive exposure to task-specific load carriage, ankle plantarflexor capacity for push-off, eccentric quadriceps and hamstring strength, hip–knee coordination, and landing or deceleration control. From an injury-prevention perspective, repeated ladder or gas-cylinder sprint drills should be scheduled with sufficient recovery, particularly when firefighters are globally fatigued, to limit repeated exposure to elevated knee demand. Because asymmetric hand-carried and shoulder-borne loads may also challenge trunk and upper-limb control, trunk stability and grip-related load-control training may be useful complementary components. However, these practical recommendations should be interpreted cautiously, as injury occurrence and upper-limb or posterior trunk-extensor activity were not directly measured in the present study [4,30,31].

### 4.5. Limitations and Future Research

Several limitations should be acknowledged. First, only healthy male firefighters were included; therefore, the results may not generalize to female firefighters, older firefighters, or those with a recent musculoskeletal injury. The absence of female firefighters is particularly important. Female firefighters may experience different relative loading because the same absolute rescue loads can represent a larger percentage of body mass and maximal strength. Sex-related differences in strength capacity, fatigue resistance, pelvic and lower-limb alignment, and trunk stabilization strategies may also influence joint work redistribution during loaded sprinting. Future studies should include female firefighters and consider both absolute and body-mass-normalized rescue loads. Second, the participants did not wear full rescue uniforms or personal protective equipment during testing. Although this choice was necessary for accurate marker placement and EMG recording, it limits the ecological validity of the simulated rescue tasks. Protective clothing, boots, heat stress, smoke, stair negotiation, turning, obstacles, restricted visibility, and real-time pressure may further alter mobility, trunk control, and lower-limb loading. Third, because load magnitude, object geometry, and carriage mode differed across the simulated rescue tasks, the present design cannot fully isolate the independent effect of load mass from task-specific handling constraints. Fourth, although task order was randomized and all rescue sprinting trials were completed as soon as possible after the fatigue criterion was reached, fatigue level may have partially recovered or accumulated across the four tasks within each fatigue condition. Future studies should verify fatigue status repeatedly between tasks.

Fifth, because only the dominant limb was analyzed, potential bilateral asymmetries induced by hand-carried or shoulder-borne loads could not be evaluated. Sixth, joint work contribution was calculated using absolute stance-phase work; this approach captures the overall mechanical work distribution but does not distinguish between energy absorption and generation. Seventh, metabolic power, oxygen uptake, and muscle oxygenation were not measured; therefore, mechanical work redistribution cannot be directly interpreted as metabolic cost. Finally, only the rectus abdominis was recorded among the trunk muscles, and no upper-limb or posterior trunk-extensor muscles were measured. Therefore, this study cannot determine how gripping, shoulder stabilization, or back-extensor activation contributed to object control and trunk–lower-limb coupling. Future studies should combine lower-limb mechanics with multi-channel EMGs of the trunk and upper limbs to clarify whole-body load-control strategies during rescue sprinting.

## 5. Conclusions

Simulated rescue sprinting under progressively heavier task-specific loads results in a redistribution of mechanical work from the ankle to the knee in male firefighters. This compensatory strategy may enable firefighters to complete high-load tasks, yet it also elevates knee mechanical demand, especially under treadmill-induced global locomotor fatigue. By extending research on load manipulation, sprint assessment, and joint energetics to a tactical occupational population, the present findings suggest that firefighter training should integrate load management, fatigue monitoring, ankle plantarflexor conditioning, knee extensor resilience, hip–knee coordination, and trunk stability training.

## Figures and Tables

**Table 1:** Participant characteristics of male firefighters (n = 53).

Variable	Mean ± SD
Age, years	27.32 ± 3.81
Height, cm	177.67 ± 3.64
Body mass, kg	75.58 ± 8.48
Firefighting service, years	4.35 ± 2.32

**Table 2:** Primary outcomes across simulated rescue-task conditions.

Outcome	Unloaded	7 kg Hose	20 kg Gas Cylinder	30 kg Fire Ladder
Stance time, s	0.138 ± 0.013	0.144 ± 0.014	0.173 ± 0.017 ^ab^	0.199 ± 0.018 ^abc^
Sprinting speed, m/s	6.112 ± 0.419	5.893 ± 0.398 ^a^	5.334 ± 0.358 ^ab^	4.871 ± 0.321 ^abc^
Peak vGRF, N/kg	28.106 ± 2.955	29.420 ± 3.307	29.737 ± 4.357	30.911 ± 5.390 ^a^
Peak knee extension moment, Nm/kg	3.98 ± 0.70	4.27 ± 0.67 ^a^	4.55 ± 0.66 ^ab^	4.71 ± 0.70 ^ab^
Hip work contribution	0.256 ± 0.055	0.262 ± 0.060	0.265 ± 0.052	0.270 ± 0.065
Knee work contribution	0.245 ± 0.061	0.273 ± 0.063 ^a^	0.347 ± 0.068 ^ab^	0.377 ± 0.064 ^abc^
Ankle work contribution	0.499 ± 0.074	0.465 ± 0.077 ^a^	0.388 ± 0.067 ^ab^	0.353 ± 0.059 ^abc^
Rectus femoris RMS	1.489 ± 1.078	1.761 ± 1.333	2.044 ± 1.653 ^a^	2.308 ± 1.755 ^ab^
Rectus abdominis RMS	1.557 ± 1.801	1.098 ± 0.997 ^a^	1.011 ± 1.216 ^a^	1.172 ± 1.127 ^a^

Values are presented as mean ± SD. Superscript letters indicate significant Bonferroni-adjusted pairwise differences between task conditions: ^a^ significantly different from unloaded sprinting; ^b^ significantly different from the 7 kg fire-hose task; and ^c^ significantly different from the 20 kg gas-cylinder task. RMS values were normalized to MVC. SD, standard deviation; vGRF, vertical ground reaction force; RMS, root mean square.

**Table 3:** Primary outcomes across fatigue states.

Outcome	Non-Fatigue	Whole-Body Fatigue	Mild Local Fatigue	Moderate Local Fatigue	Severe Local Fatigue
Stance time, s	0.163 ± 0.030	0.165 ± 0.030	0.161 ± 0.028 ^B^	0.164 ± 0.028 ^C^	0.164 ± 0.028 ^C^
Sprinting speed, m/s	5.611 ± 0.691	5.477 ± 0.609 ^A^	5.614 ± 0.595 ^B^	5.534 ± 0.577 ^AC^	5.527 ± 0.585 ^AC^
Peak vGRF, N/kg	29.870 ± 5.022	29.183 ± 3.810	29.709 ± 4.204	29.601 ± 3.828	29.355 ± 4.204
Peak knee extension moment, Nm/kg	4.32 ± 0.68	4.51 ± 0.79 ^A^	4.40 ± 0.74	4.41 ± 0.72	4.25 ± 0.74 ^BCD^
Hip work contribution	0.270 ± 0.053	0.255 ± 0.059 ^A^	0.270 ± 0.070 ^B^	0.259 ± 0.054 ^C^	0.261 ± 0.055
Knee work contribution	0.302 ± 0.080	0.325 ± 0.086 ^A^	0.306 ± 0.078 ^B^	0.311 ± 0.086	0.310 ± 0.085 ^B^
Ankle work contribution	0.428 ± 0.091	0.420 ± 0.091	0.424 ± 0.084	0.430 ± 0.096	0.429 ± 0.091
Rectus femoris RMS	2.330 ± 1.740	1.900 ± 1.269 ^A^	1.851 ± 1.448 ^A^	1.825 ± 1.572 ^A^	1.599 ± 1.403 ^A^
Rectus abdominis RMS	1.209 ± 1.079	1.969 ± 2.200 ^A^	0.970 ± 0.728 ^AB^	0.903 ± 0.815 ^AB^	0.997 ± 1.011 ^AB^

Values are presented as mean ± SD. Superscript letters indicate significant Bonferroni-adjusted pairwise differences between fatigue states: ^A^ significantly different from non-fatigue; ^B^ significantly different from whole-body fatigue; ^C^ significantly different from mild local fatigue; and ^D^ significantly different from moderate local fatigue. RMS values were normalized to MVC. SD, standard deviation; vGRF, vertical ground reaction force; RMS, root mean square.

**Table 4:** ANOVA results and effect sizes for outcomes reported in the Results Section.

Outcome	Effect	F	*p*	ηp^2^
Stance time, s	Fatigue	3.682	0.009	0.066
	Task	260.759	<0.001	0.834
	Fatigue × task	2.088	0.022	0.039
Sprinting speed, m/s	Fatigue	16.338	<0.001	0.239
	Task	162.750	<0.001	0.758
	Fatigue × task	5.848	<0.001	0.101
Step width, m	Fatigue	1.945	0.101	0.036
	Task	3.796	0.011	0.068
	Fatigue × task	1.842	0.038	0.034
Peak hip flexion angle, deg	Fatigue	4.190	0.004	0.075
	Task	29.970	<0.001	0.366
	Fatigue × task	1.394	0.175	0.026
Peak knee flexion angle, deg	Fatigue	3.426	0.100	0.062
	Task	26.619	<0.001	0.339
	Fatigue × task	1.745	0.057	0.032
Peak ankle dorsiflexion angle, deg	Fatigue	20.767	<0.001	0.285
	Task	22.225	<0.001	0.299
	Fatigue × task	0.694	0.758	0.013
Peak knee extension angle magnitude, deg	Fatigue	6.346	<0.001	0.109
	Task	3.435	0.018	0.062
	Fatigue × task	2.242	0.013	0.041
Vertical CoM displacement, m	Fatigue	6.331	<0.001	0.109
	Task	70.798	<0.001	0.577
	Fatigue × task	0.988	0.457	0.019
Lateral CoM displacement, m	Fatigue	8.815	<0.001	0.145
	Task	0.467	0.705	0.009
	Fatigue × task	8.174	0.013	0.136
Peak vGRF, N/kg	Fatigue	2.120	0.081	0.039
	Task	6.445	<0.001	0.110
	Fatigue × task	2.330	0.008	0.043
Peak knee extension moment, Nm/kg	Fatigue	7.635	<0.001	0.128
	Task	22.357	<0.001	0.301
	Fatigue × task	0.792	0.650	0.015
Peak knee flexion moment magnitude, Nm/kg	Fatigue	2.126	0.079	0.039
	Task	0.287	0.835	0.005
	Fatigue × task	2.669	0.002	0.049
Hip work contribution	Fatigue	5.482	<0.001	0.095
	Task	0.854	0.466	0.016
	Fatigue × task	2.630	0.004	0.048
Knee work contribution	Fatigue	6.900	<0.001	0.117
	Task	95.958	<0.001	0.649
	Fatigue × task	2.104	0.016	0.039
Ankle work contribution	Fatigue	1.590	0.175	0.030
	Task	80.809	<0.001	0.608
	Fatigue × task	1.859	0.036	0.035
Rectus femoris RMS	Fatigue	10.867	<0.001	0.173
	Task	6.335	<0.001	0.109
	Fatigue × task	1.185	0.295	0.022
Rectus abdominis RMS	Fatigue	30.546	<0.001	0.370
	Task	6.056	<0.001	0.104
	Fatigue × task	2.741	0.018	0.050

Results are from two-way 5 × 4 repeated-measures ANOVA testing the main effects of fatigue state and rescue task and their interaction. Greenhouse–Geisser-corrected *p* values were reported when the sphericity assumption was violated. Effect sizes are reported as partial eta squared. ANOVA, analysis of variance; ηp^2^, partial eta squared; vGRF, vertical ground reaction force; RMS, root mean square; CoM, center of mass.

## Data Availability

The data presented in this study are available from the corresponding author upon reasonable request.

## Abbreviations

The following abbreviations are used in this manuscript:

MVC	Maximum Voluntary Contraction
EMG	Electromyography
RMS	Root Mean Square
vGRF	vertical Ground Reaction Force
CoM	Center of Mass
ηp^2^	Partial Eta Squared
